# Task-Based and Questionnaire Measures of Inhibitory Control Are Differentially Affected by Acute Food Restriction and by Motivationally Salient Food Stimuli in Healthy Adults

**DOI:** 10.3389/fpsyg.2016.01303

**Published:** 2016-08-29

**Authors:** Savani Bartholdy, Jiumu Cheng, Ulrike Schmidt, Iain C. Campbell, Owen G. O'Daly

**Affiliations:** ^1^Section of Eating Disorders, Department of Psychological Medicine, Institute of Psychiatry, Psychology and Neuroscience, King's College LondonLondon, UK; ^2^Department of Neuroscience, Institute of Psychiatry, Psychology and Neuroscience, King's College LondonLondon, UK; ^3^Centre for Neuroimaging Sciences, Institute of Psychiatry, Psychology and Neuroscience, King's College LondonLondon, UK

**Keywords:** inhibitory control, temporal discounting, food, fed, fasted

## Abstract

Adaptive eating behaviors are dependent on an interaction between motivational states (e.g., hunger) and the ability to control one's own behavior (inhibitory control). Indeed, behavioral paradigms are emerging that seek to train inhibitory control to improve eating behavior. However, inhibitory control is a multifaceted concept, and it is not yet clear how different types (e.g., reactive motor inhibition, proactive motor inhibition, reward-related inhibition) are affected by hunger. Such knowledge will provide insight into the contexts in which behavioral training paradigms would be most effective. The present study explored the impact of promoting a “need” state (hunger) together with motivationally salient distracting stimuli (food/non-food images) on inhibitory control in 46 healthy adults. Participants attended two study sessions, once after eating breakfast as usual and once after acute food restriction on the morning of the session. In each session, participants completed questionnaires on hunger, mood and inhibitory control, and undertook task-based measures of inhibitory control, and had physiological measurements (height, weight, and blood glucose) obtained by a researcher. Acute food restriction influenced task-based assessments but not questionnaire measures of inhibitory control, suggesting that hunger affects observable behavioral control but not self-reported inhibitory control. After acute food restriction, participants showed greater temporal discounting (devaluation of future rewards), and subjective hunger and these were inversely correlated with stop accuracy on the stop signal task. Finally, participants generally responded faster when food-related distractor images were presented, compared to non-food images, independent of state. This suggests that although food stimuli motivate approach behavior, stimulus relevance does not impact inhibitory control in healthy individuals, nor interact with motivational state. These findings may provide some explanation for poorer inhibitory control often reported in studies of individuals who practice restraint over eating.

## Introduction

We live in an obesogenic environment. Thus, we need to be able to control our eating behavior and overcome the temptation toward unhealthy foods that are often readily accessible, or to avoid non-homeostatic eating (i.e., eating in the absence of a physiological energy deficit). The ability to adjust our behavior to adapt to our environment depends on the ability to stop/withhold inappropriate behaviors (broadly termed “inhibitory control”), as well as the ability to determine the salience and importance of environmental cues. This includes approaching items that fulfill a basic need or have rewarding properties (e.g., food), but only when this is contextually appropriate (i.e., when hungry compared to when satiated).

Highly palatable and often calorific foods are easily accessible and the ability to exercise inhibitory control is crucial to resisting them. Inhibitory control is a multifaceted concept, and different types of inhibitory control may be relevant in such a context. For example, one needs to exercise proactive and reactive motor response inhibition (i.e., withholding a motor response in the context of uncertainty, or in reaction to a stop signal, respectively) to stop from purchasing and/or eating such foods. Similarly, reward-related inhibition (i.e., waiting for a larger delayed reward rather than choosing immediate gratification) is required to overcome the temptation of, for example, a highly calorific snack and wait to eat a more substantial meal when hungry. Thus, adaptive eating behaviors are dependent on an interaction between motivational/physiological states (e.g., hunger) and inhibitory control. Indeed, neuroimaging studies have revealed that brain activity in response to food stimuli differs according to hunger state, with satiety associated with a relatively reduced response in reward-related regions and an enhanced response in regions implicated in executive control in healthy individuals compared to a pre-meal state (Thomas et al., 2015), suggesting that the neural correlates of inhibitory control in the context of food is associated with hunger state.

Altered inhibitory control has been proposed as a key factor in the aetiopathology of eating disorders and obesity (e.g., Brooks et al., 2012; Thamotharan et al., 2013; Berner and Marsh, 2014; Wierenga et al., 2014). There is evidence of reduced reward-related inhibitory control in individuals with bulimia nervosa, binge eating disorder and obesity (Davis et al., 2010; Manwaring et al., 2011; Galimberti et al., 2012; Mole et al., 2015; Kekic et al., 2016), and the opposite in anorexia nervosa (Steinglass et al., 2012; Decker et al., 2015) compared to healthy individuals (for review, see McClelland et al., In prep.). Similar findings have also been found with respect to reactive inhibition, although the findings have been less consistent than data on reward-related inhibition (for review, see Bartholdy et al., 2016). As a result, behavioral paradigms are emerging that seek to increase inhibitory control to improve eating behavior (Lawrence et al., 2015; for reviews, see Brockmeyer et al., 2015; Bartholdy et al., 2016; Turton et al., 2016), which may be useful both in the treatment of eating disorders/obesity, but also as possible preventative measures for individuals in the community. However, the relationships between hunger and the different elements of inhibitory control are not yet clear. For example, does being in a state of hunger affect all elements of inhibitory control affected in the same way? This information will be important in determining the contexts in which such training should be provided to improve each of the types of inhibitory control.

There have been some studies exploring the relationship between hunger and inhibitory control. A relationship between hunger state and inhibitory control has also been reported in behavioral experiments. Nordgren et al. (2009) reported that hungry individuals had a weaker belief in their ability to control their impulses than satiated individuals and exposed themselves to less temptation (preferred snacks), argued to be a result of the weakened beliefs in their impulse-control abilities. In healthy overweight and obese women, temporal discounting (i.e., the devaluation of rewards over time) and hedonic hunger (i.e., a preoccupation with palatable foods or desire to eat for pleasure in the absence of a physical energy deficit; Witt et al., 2014; Manasse et al., 2015) were found to interact to predict food intake only in individuals high in temporal discounting (Appelhans et al., 2011; Manasse et al., 2015), suggesting that a greater ability to delay reward (i.e., lower temporal discounting) may be protective of overeating. Moreover, there is evidence that in healthy individuals, being hungry is associated with reduced temporal discounting but only for large rewards (De Ridder et al., 2014), which the authors argued may be due to increased reliance on emotion or intuition in decision making when in a motivational physical state. Reactive motor response inhibition in healthy individuals, however, does not appear to be influenced by hunger: one study reported no correlation between inhibitory control and hunger (Haynes et al., 2015), and another reported that the effect of inhibitory control on food intake was not affected by hunger (before/after lunch; Nederkoorn et al., 2015). However, reactive motor response inhibition has been reported to *interact* with hunger, with healthy individuals who are both hungry and more impulsive (i.e., have less inhibitory control) consuming the greatest number of calories in a subsequent taste test, and purchased more snack calories in a virtual supermarket, compared to participants who were either hungry or impulsive, separately (Nederkoorn et al., 2009). If hunger and inhibitory control interact to influence food intake, then it can be predicted that training paradigms aimed at improving inhibitory control would be most beneficial if completed in the context of hunger. However, while the above studies imply a relationship between hunger and inhibitory control, the employment of between-subjects designs only permits cross-sectional inferences to be drawn, as each participant was only assessed once at a particular level of hunger. It is important to replicate these findings using repeated measures designs to determine how hunger can affect inhibitory control within the same individual. This will improve our understanding of why some individuals may experience difficulty in exercising control over their eating behavior, and will provide insight into the contexts in which behavioral interventions targeting inhibitory control over eating would be most effective.

In relation to contextual effects, it is important to explore whether motivationally salient stimuli additionally affect one's ability to exercise inhibitory control. For example, one study found that response times on a simple reaction time task correlated with a greater desire to eat when participants were asked to simultaneously imagine their favorite food, but this relationship was not observed when participants imagined a non-food-related scenario (Green et al., 2000). Food is a primary reinforcer, is typically appetitive and generates an approach-motivated emotional state (Rolls, 1999; Uher et al., 2014). Motivational salience attributed to food stimuli is easily manipulated by asking participants to abstain from eating. Thus, in a food deprived (fasted) state, food appears more pleasant, elicits less disgust, facilitates approach reactions, and is associated with enhanced startle reflexes and increased heart rate (Drobes et al., 2001; Seibt et al., 2007; Hoefling et al., 2009). In addition, increased attentional bias toward food has been demonstrated in people who are hungry/fasted (Castellanos et al., 2009) or are sensitive to external food cues (Brignell et al., 2009). Motivational state (i.e., hunger vs. satiety) may therefore modulate the personal relevance of food stimuli and have an impact on the salience of state-relevant cues (Oliveira et al., 2013).

This present study investigated the influence of promoting a “need” state (hunger) and motivationally relevant distraction (food images) on several aspects of inhibitory control. Specifically, it examined the impact of acute food-restriction on questionnaire and task-based measures of inhibitory control, including proactive, reactive, and reward-based inhibition, using a repeated-measures design. In addition, the motivational relevance of distracting stimuli (food vs. non-food images) on proactive inhibition and reactive inhibition was investigated. It was broadly hypothesized that (a) acute food restriction would impair inhibitory control, (b) the presence of food stimuli compared to neutral (non-food) stimuli would promote “approach” behaviors resulting in poorer inhibitory control, and (c) a hunger state and the motivational relevance of the food images would interact, with the poorest inhibitory control observed on trials including food images in the fasted state.

## Materials and methods

### Participants

Fifty seven healthy adults were recruited from King's College London for the study (31 students, 14 employed staff, 1 unemployed). Four participants met DSM-IV criteria for an eating disorder on the Eating Disorder Diagnostic Scale (Stice et al., 2000) and were therefore excluded from the analysis. One participant did not follow the study instructions during the first session so did not complete the second session. An estimate of weight and height was self-reported by participants during screening, however this was discrepant with the weight obtained during the study, with three participants being underweight (BMI < 18.5 kg/m^2^) and four participants being overweight (BMI > 25 kg/m^2^). As weight status may affect inhibitory control (Bartholdy et al., 2016), these individuals were excluded from analyses for a more homogeneous sample. The final sample consisted of 46 participants (39 women), with a mean age of 24.9 years (SD = 6.62 years, range = 18–49 years) and mean BMI (average across both sessions) of 21.6 kg/m^2^ (SD = 1.69 kg/m^2^). The majority (87.0%) of participants were right handed. Exclusion criteria included being under 18 years of age, a past or current axis I mental disorder, neurological disease, history of head trauma with loss of consciousness, current use of psychotropic medication, current use of illicit drugs, and drinking on average >18 units of alcohol per week.

### Computer tasks

#### Temporal discounting task

TD refers to the tendency of people to discount (reduce the magnitude of) the value of future rewards with increasing distance from the present. This was assessed using a hypothetical monetary choice task, in which participants were asked to indicate their preference between a smaller immediate reward (smaller-sooner reward) and a larger delayed reward (larger-later reward) on 80 binary choices, modeled on a paradigm developed by Steinglass et al. (2012). A monetary choice task was used to explore the degree to which any impact of hunger on inhibitory control generalized outside of the context of food. Greater discounting refers to an increased preference for smaller-sooner rewards.

The rewards were presented using two decision frames: an Accelerate frame in which the smaller-sooner reward varied between £20 and £98 in £2 increments (40 choices) while the larger-later reward remained fixed at £100, and a Delay frame in which the smaller-sooner reward remained at £50 while the larger-later reward varied between £52 and £130 in £2 increments (40 choices). The larger-later delay was 3 months (0.25 years) for all choices. The paradigm involved a single block including all 80 trials, presented in a randomized order, however the order of trials was kept consistent across all participants.

Discount rate was determined by calculating participants' discount factor, i.e., the degree to which the present subjective value of a future reward is reduced. This was calculated for each set (Accelerate, Delay) and a global DF was calculated as the mean discount factor of the two sets. Discount factors were quantified using a two-step procedure (Steinglass et al., 2012). The first step establishes the “indifference point” for that set, i.e., the point at which choices are considered equivalent. This is identified as the choice where the participant's preference switches from larger-later to smaller-sooner in the Accelerate set, and from smaller-sooner to larger-later in the Delay set (Steinglass et al., 2012). Second, the following formula is fit to the indifference point to calculate the discount factor: δ = (*x*_1_/*x*_2_)^(1/(*t*_2_−*t*_1_))^, where *x*_1_ is the smaller-sooner reward, *x*_2_ is the larger-later reward, and *t*_2_ − *t*_1_ is the delay to reward presentation (in years), which was 0.25 in this study (Weber et al., 2007; Steinglass et al., 2012). This procedure is a sensitive measure of temporal discounting that is independent of hyperbolic modeling and area under the curve analyses (Weber et al., 2007). The discount factor value ranges from 0 to 1, with smaller numbers indicating greater temporal discounting and thus a greater tendency to choose the smaller-sooner reward. In the case that no switch was made (i.e., participants consistently chose either the smaller-sooner or larger-later reward across all choices), the indifference point was unable to be calculated and therefore a respective default score of 0 (consistent smaller-sooner selection) or 1 (consistent larger-later selection) was assigned.

#### Modified proactive inhibition task (PI)

This task was a modified version of a simple cued-reaction time task based on previous paradigm designs used to assess proactive inhibition (Jaffard et al., 2008), in which the delay between “warning” cue and target onset was varied across trials. An image flanked by two empty boxes was presented on a computer screen. On each trial, a target (large yellow dot) appeared in one of the two boxes. Participants are instructed to react as quickly as possible to the location of the visual target by pressing the corresponding arrow key. On some trials, the target was preceded by a warning signal (“cued trials” compared to “non-cued trials”), which appeared in both boxes simultaneously and was consequently spatially uninformative.

This study employed a blocked design. The image in the center of the screen changed on each trial, and consisted of either a high/low calorie food image, a neutral non-food image (household items such as stationary), or a fixation cross. The order of image presentation was randomized, with an equal number of each image type presented. The stimulus onset asynchrony was manipulated so that the cue-target delay (stimulus-onset asynchrony; SOA) varied randomly across four conditions: 0 (no cue), 100, 300, and 500 ms. This study was conducted in three blocks: one “pure” block (only non-cued trials) and two mixed blocks in which cued- and non-cued trials were presented randomly. Participants completed a practice block of 18 trials. Participants then completed three experimental blocks in a randomized, counterbalanced order. One block consisted only of non-cued trials (“pure” block; 63 trials). The SOA did not vary in the pure block. The remaining two blocks included a mixture of non-cued (0 ms SOA) and cued trials (at varying SOAs: 100, 300, 500 ms). A total of 63 non-cued trials and 63 cued trials (21 trials at each SOA) were presented in a random order across the mixed blocks. The playlists for each block were the same for all participants in all sessions. Across all blocks, the inter-trial interval was 3000 ms, cue duration was 300 ms and target onset (from start of trial) was 2000 ms.

Two behavioral indices were calculated for entry into correlational analyses. The first was an index of the overall benefit gained from warning cues (“Warning benefit”), calculated by subtracting the mean reaction time (RT) on trials with the longest delay between cue and target (500 ms SOA) from the non-cued trials (0 ms SOA in the mixed blocks). The second was an index of proactive slowing in the context of uncertainty, calculated by subtracting the mean RT on trials in the pure block from non-cued trials in the mixed block (“Preparation cost”; Chikazoe et al., 2009). Faster reaction time during the pure block compared to 0 ms SOA trials in the mixed blocks should indicate that participants implicitly recognized a difference in the probability that the visual stimulus was the target, i.e., their threshold for responding is altered.

#### Modified stop signal task

The stop signal task is a reaction time task in which participants are asked to respond to one or more “go” stimuli. On each trial, participants were presented with a go signal in the form of a left- or right-pointing arrow in the direction of the required response. On 20% of trials, a stop signal (red dot) was presented at irregular intervals in order to minimize predictability. Following the presentation of this stop signal, participants were required to inhibit their response to the go signal. The delay between the go signal and stop signal is termed the stop signal delay (SSD). The ability to inhibit a response is dependent on the length of the SSD: the longer the delay, the harder it is to inhibit a response. The SSD was varied from trial to trial by 50 ms increments (ranging between 150 and 900 ms) in a staircase procedure, which intended to converge subjects toward an overall performance of 50% for each run. The initial SSD was set at 150 ms. The primary outcome variable of the stop signal task is the stop signal reaction time (SSRT), which is considered an index of inhibitory control ability. Typically, this is calculated by subtracting the SSD from the mean RT on go trials. However, due to the variability in stop accuracy and the small number of trials in this design, the SSRT in the present study was calculated by subtracting the mean SSD on stop trials from the nth percentile of the correct go RT distribution, using the formula: SSRT = RT (m)−SSD, where m = n(number of correct go responses) ^*^ probability_(responding|signal)_ (Nederkoorn et al., 2012). In other words, if participants correctly stopped on 25% of the trials, the mean SSD was subtracted from the 25th% reaction time on correct go trials [0.25^*^n_(correct go trials)_] to calculate the SSRT.

This study employed a blocked design. Participants first completed a practice block of 20 trials including 8 stop trials. This was followed by three experimental blocks of 100 trials each (20 stop trials): two blocks where irrelevant distractor stimuli (one block with high/low calorie food images, one block with neutral non-food images [e.g., household objects]) were presented beside the arrow (on the same side of the screen as the arrow was pointing to minimize congruency-related interference effects), and one block with no additional stimuli (no image condition). The blocks were presented in a randomized, counterbalanced order across participants, and the order of stop and go trials within each block was randomized. The playlists for each block were the same for all participants in all sessions. The arrow pointed to the left and right on an equal number of trials. On all trials, the arrow display duration (unless cut short by a stop signal) was 400 ms, stop signal duration was 300 ms. The maximum RT from the onset of the arrow was 1400 ms. The inter-trial interval was fixed at 1800 ms.

### Stimuli

The food and non-food images were taken from an in-house image battery. standardized for size, resolution, color luminance, and complexity (Karra et al., 2013).

### State manipulation (fed/fasted)

As has been done previously (e.g., Karra et al., 2013; Chechko et al., 2015), hunger was manipulated via an overnight fast. Adherence to the overnight fast was assessed via open-ended questions regarding time since last eaten (including drinks), what food/drink was last eaten, and the time since last consumption of a caffeinated beverage (Witt et al., 2014).

A small blood sample was obtained through a finger prick to assess the difference in blood glucose in the fed and fasted state, as used by patients with diabetes for self-monitoring of blood glucose (for review, see Clarke and Foster, 2012).

### Procedure

Individuals who expressed interest in the study completed a telephone screening to assess eligibility using a study-specific screening questionnaire developed by the researchers, including questions about their diet, substance use, caffeine intake, smoking, and history of neurological trauma and psychiatric disorder. Participants were asked to estimate their weight and height in order for the researchers to get an estimated BMI for assessment of eligibility.

Eligible participants attended two experimental sessions occurring at the same time of day on 2 separate days spaced no more than 1 week apart. Participants were asked to fast (i.e., refrain from eating or drinking anything except water) on the morning of one of the study sessions and eat breakfast as usual for the other study session. The order of study conditions was randomized and counterbalanced across participants (54.7% fed and 45.3% fasted first condition).

At both study sessions, participants were first asked to complete several questionnaires [Depression, Anxiety, and Stress Scale (Lovibond and Lovibond, 1995), Barratt Impulsiveness Scale (Patton et al., 1995), Delaying Gratification Inventory (Hoerger et al., 2011)]. Hunger was assessed using a 10 cm visual analog scale. Participants were also asked to state when they last ate, what their last meal consisted of, and when they next expected to eat. In the first session, participants additionally completed the Eating Disorder Examination Questionnaire (Fairburn, 2008) and the screening module of the Structured Clinical Interview for DSM-IV (First et al., 2002). Participants then engaged in three computerized tasks: the Temporal Discounting Task, the Stop Signal Task, and a simple cued-reaction time task assessing proactive inhibition. The order in which the tasks were completed was randomized and counterbalanced across participants and across sessions. After completing the tasks, the researcher measured the participants' height and weight, blood glucose level and temperature. Participants were thanked and received £20 compensation for their time and any additional reimbursement for travel.

### Ethics

This study was reviewed and approved by the Psychiatry, Nursing, and Midwifery Research Ethics Subcommittee at King's College London (PNM/13/14-147). All participants gave written consent after the procedures were explained and were debriefed after the experiment.

### Data analysis

Data was collected using in-house software and analyzed using SPSS and were corrected for multiple comparisons using Bonferroni correction. Statistical analyses were performed using IBM® SPSS® software (Version 22). All tests were two-tailed and the level of significance was set at α = 0.05.

#### Questionnaires

Inspection of histograms, skewness and kurtosis indicated that the majority of physiological and questionnaire data (hunger, temperature, time to next meal, and time since last meal) were skewed. Glucose, DGI total and physical subscales, and the BIS-11 attention subscale were the only questionnaire and physiological measures that did not show skewness or kurtosis in either the fed or fasted state. With respect to the physiological data, this was unsurprising: hunger and time since last meal were positively skewed in the fed state only, as participants were asked to ensure they had eaten breakfast as usual that day. Similarly, time to next meal in the fasted state was positively skewed. Thus, Wilcoxon Signed Rank tests were employed to compare questionnaire responses and physiological outcomes in the fed and fasted state. These tests were corrected for multiple comparisons using Bonferroni correction.

#### Temporal discounting task

While Global discount factor was normally distributed, Accelerate discount factor and Delay discount factor were positively skewed. Square root transformations were effective in normalizing the temporal discounting data. The main effects of and interaction between state and frame were analyzed using a repeated measures 2 × 2 (state × frame) ANOVA.

#### Proactive inhibition task

Gradual adjustment of behavioral inhibition was assessed using 2 × 3 × 4 ANOVA to evaluate the main effects and interactions between state (fed/fasted), stimulus (food/non-food/fixation cross) and SOA (0, 100, 300, 500 ms) on reaction time. The effect of uncertainty on non-cued trials was assessed using a 2 × 2 × 3 repeated measures to examine the main effects and interactions of block condition (pure vs. mixed), state and stimulus on reaction time. *Post-hoc t*-tests corrected for multiple comparisons using Bonferroni correction were employed to further assess statistically significant main effects and interactions. The 2 indices of proactive inhibition (warning benefit, preparation cost) were entered into correlational analyses (below).

#### Stop signal task

Mean RT and SSRT data from the stop signal task were also positively skewed. Square-root transformations were effective in normalizing the stop signal task data. Go accuracy could not be normalized through transformations due to almost perfect accuracy. Repeated measures 2 × 3 ANOVAs were conducted on the SSRT, mean RT and stop accuracy data to explore the main effects of and interactions between state (fed/fasted) and stimulus (food/non-food/no image). Statistically significant main effects were further explored using *post-hoc t*-tests corrected for multiple comparisons using Bonferroni correction.

#### Correlations

Correlations were conducted to assess the relationship between physiological measures, mood, and questionnaire and task-based measures of inhibitory control across states. In situations where a main effect of state is observed on both variables entered into the correlation, the relationship between the variables were assessed separately for the fed and fasted sessions due to the increased likelihood of spurious correlations associated with clustering due to state effects. Pearson's r and Spearman's rho tests were employed when data were parametric and non-parametric, respectively.

## Results

### State manipulation

The length of fasting time in the fasted condition ranged from 8 to 17 h. To assess whether our manipulation of hunger state was successful, paired samples *t*-tests were conducted to examine differences in self-reported hunger, time since last meal and time to next meal and blood glucose in the fed compared to fasted state (Table 1). All comparisons were statistically significant after Bonferroni correction for multiple comparisons. Participants were significantly more hungry in the fasted condition [*z*_(33)_ = 5.012, *p* < 0.001], had lower blood glucose levels [*z*_(46)_ = −2.918, *p* = 0.004], had a longer time since last eating [*z*_(42)_ = 5.639, *p* < 0.001] and a shorter intended waiting time before the next meal [*z*_(42)_ = −3.921, *p* < 0.001] in the fasted compared to fed condition.

**Table 1:** **Differences in state measurements, questionnaire responses, and temporal discounting between fed and fasted states**.

	**Fed Mean(SD)**	**Fasted Mean(SD)**	***Wilcoxon z statistic***	***N***	***p*^a^**
**STATE MEASUREMENTS**					
Hunger	0.2 (0.15)	0.6 (0.17)	5.012	33	<0.001
Glucose (mmol/liter)	5.1 (0.59)	4.8 (0.47)	−2.918	46	0.004
Time since last meal (hours)	1.9 (1.80)	12.8 (2.00)	5.639	42	<0.001
Time to next meal (hours)	2.4 (0.95)	1.5 (0.70)	−3.921	42	<0.001
**DEPRESSION, ANXIETY, AND STRESS SCALE (DASS)**					
Total	6.2 (4.90)	6.3 (4.94)	1.061	46	0.289
Depression	1.5 (1.52)	1.4 (1.50)	−0.698	46	0.485
Anxiety	1.4 (1.89)	1.3 (1.48)	0.212	46	0.832
Stress	3.3 (2.50)	3.6 (3.08)	0.858	46	0.391
**DELAYING GRATIFICATION INVENTORY (DGI)**					
Total	136.4 (12.47)	134.5 (12.32)	−1.717	45	0.086
Achievement	29.5 (3.85)	29.3 (4.33)	−0.459	45	0.646
Food	24.9 (4.42)	24.5 (4.73)	−0.657	45	0.511
Money	29.1 (4.38)	28.6 (4.37)	−1.481	45	0.139
Physical	25.7 (3.64)	25.1 (3.95)	−1.396	45	0.163
Social	27.3 (2.87)	27.0 (2.92)	−0.783	45	0.434
**BARRATT IMPULSIVENESS SCALE (BIS-11)**					
Total	44.0 (9.22)	45.8 (8.27)	1.254	45	0.210
Attention	12.1 (2.86)	12.3 (2.80)	0.108	45	0.914
Motor	18.8 (4.08)	19.8 (3.49)	1.795	45	0.073
Non-planning	13.2 (4.08)	13.7 (3.82)	0.972	45	0.331

a *Uncorrected p-values*.

### Self-reported impulsivity and mood

Differences in self-reported mood and impulsivity were assessed by comparing responses on the DASS-21 and on the BIS-11 and DGI questionnaires, respectively, in the fed and fasted state using paired samples *t*-tests. There was a trend for impulsivity assessed by the DGI questionnaire (DGI Total: *z* = −1.717, *p* = 0.086) and motor impulsivity on the BIS-11 (Motor subscale score: *z* = 1.795, *p* = 0.073) to be higher in the fasted state, however this was no longer observed after correction for multiple comparisons. The analyses did not yield any other significant differences between states (Table 1, all *p* > 0.193).

### Temporal discounting

**Figure 1:**
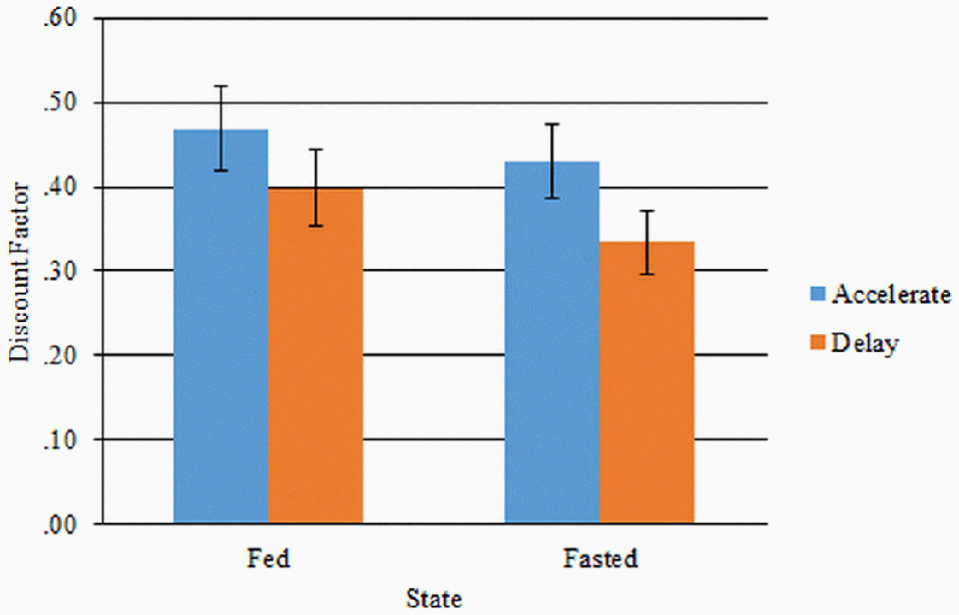
**Discount factors for the accelerate and delay frame in the fed and fasted state**. Error bars denote standard errors.

The impact of state and decision framing was assessed using a 2 × 2 repeated measures ANOVA using transformed discount factor scores (Figure 1). This analysis yielded a main effect of frame [*F*_(1, 45)_ = 11.769, *p* = 0.001] and a trend toward a main effect of state [*F*_(1, 45)_ = 3.587, *p* = 0.065], but no interaction between state and frame [*F*_(1, 45)_ = 1.375, *p* = 0.247] (Figure 1). *Post-hoc t*-tests revealed the main effect of frame was driven by lower discount factors (indicating greater temporal discounting) in the delay frame (mean = 0.55, SD = 0.232) compared to the accelerate frame (mean = 0.61, SD = 0.262), *t*_(45)_ = 3.341, *p* = 0.001. Discount factors were lower in the fasted (mean = 0.56, SD = 0.243) compared to the fed (mean = 0.60, SD = 0.255) state, however this trend did not survive correction for multiple comparisons, *t*_(45)_ = 1.894, *p* = 0.065 (uncorrected).

### Proactive inhibition

This task was completed by 31 participants (27 women). Due to computer malfunctions during the pure block in the fed state for two female participants, these participants were excluded from analyses involving pure block data. Means and standard deviations for reaction times, accuracy and the behavioral indices of proactive inhibition for each trial type in each state are described in Table 2.

**Table 2:** **Mean (standard deviation) RT and accuracy for the non-cued trials (in the pure and mixed blocks) and for each SOA (in the mixed blocks), and mean behavioral index of proactive inhibition for each stimulus type in the fed and fasted state**.

	**SOA (ms)**	**Fed**			**Fasted**		
		**Food**	**Non-food**	**Fixation**	**Food**	**Non-food**	**Fixation**
**RT (ms)**							
Non-cued trials (Pure vs. 0ms; *n* = 29)	Pure	347.71 (40.88)	357.72 (41.06)	371.95 (44.43)	352.98 (45.27)	363.84 (44.11)	380.45 (52.62)
	0	396.08 (46.10)	392.97 (35.23)	409.50 (43.00)	405.14 (54.58)	411.06 (53.94)	423.38 (53.77)
Mixed block SOA (*n* = 31)	0	396.82 (45.61)	393.05 (34.70)	410.94 (41.92)	403.25 (53.37)	409.10 (52.69)	421.54 (52.47)
	100	359.92 (43.63)	357.01 (40.18)	370.25 (37.27)	362.84 (39.88)	374.89 (40.73)	375.68 (44.60)
	300	364.34 (45.01)	371.17 (39.20)	374.14 (40.70)	374.47 (33.77)	375.83 (41.69)	387.42 (36.77)
	500	333.19 (33.47)	328.77 (45.39)	329.19 (32.63)	337.07 (51.01)	339.33 (35.22)	342.86 (34.40)
**ACCURACY (%)**							
Non-cued trials (Pure vs. 0ms; *n* = 29)	Pure	99.01 (2.34)	98.69 (2.17)	98.03 (3.25)	99.01 (2.34)	99.01 (2.34)	98.85 (2.07)
	0	99.06 (2.23)	98.97 (2.46)	99.67 (1.23)	99.37 (1.60)	98.62 (2.64)	98.85 (2.43)
Mixed block SOA (*n* = 31)	0	99.12 (2.17)	99.03 (2.39)	99.69 (1.19)	99.41 (1.55)	98.71 (2.57)	98.92 (2.37)
	100	94.01 (8.86)	97.24 (5.74)	97.24 (6.82)	94.01 (8.86)	95.85 (8.41)	97.7 (5.34)
	300	98.62 (4.29)	96.77 (7.10)	100 (0.00)	99.08 (3.57)	97.7 (6.49)	99.54 (2.57)
	500	95.85 (6.59)	98.16 (4.87)	97.7 (6.49)	97.7 (5.34)	98.16 (4.87)	96.77 (6.07)
**BEHAVIORAL INDEX (ms)**							
Warning benefit		63.63 (38.43)	64.28 (33.85)	81.75 (29.38)	66.19 (45.65)	69.77 (37.86)	78.68 (45.78)
Preparation cost		48.37 (36.88)	35.25 (26.88)	37.55 (30.28)	52.15 (38.63)	47.22 (33.21)	42.94 (30.11)

#### Effect of uncertainty in cued trials: warning benefit

**Figure 2:**
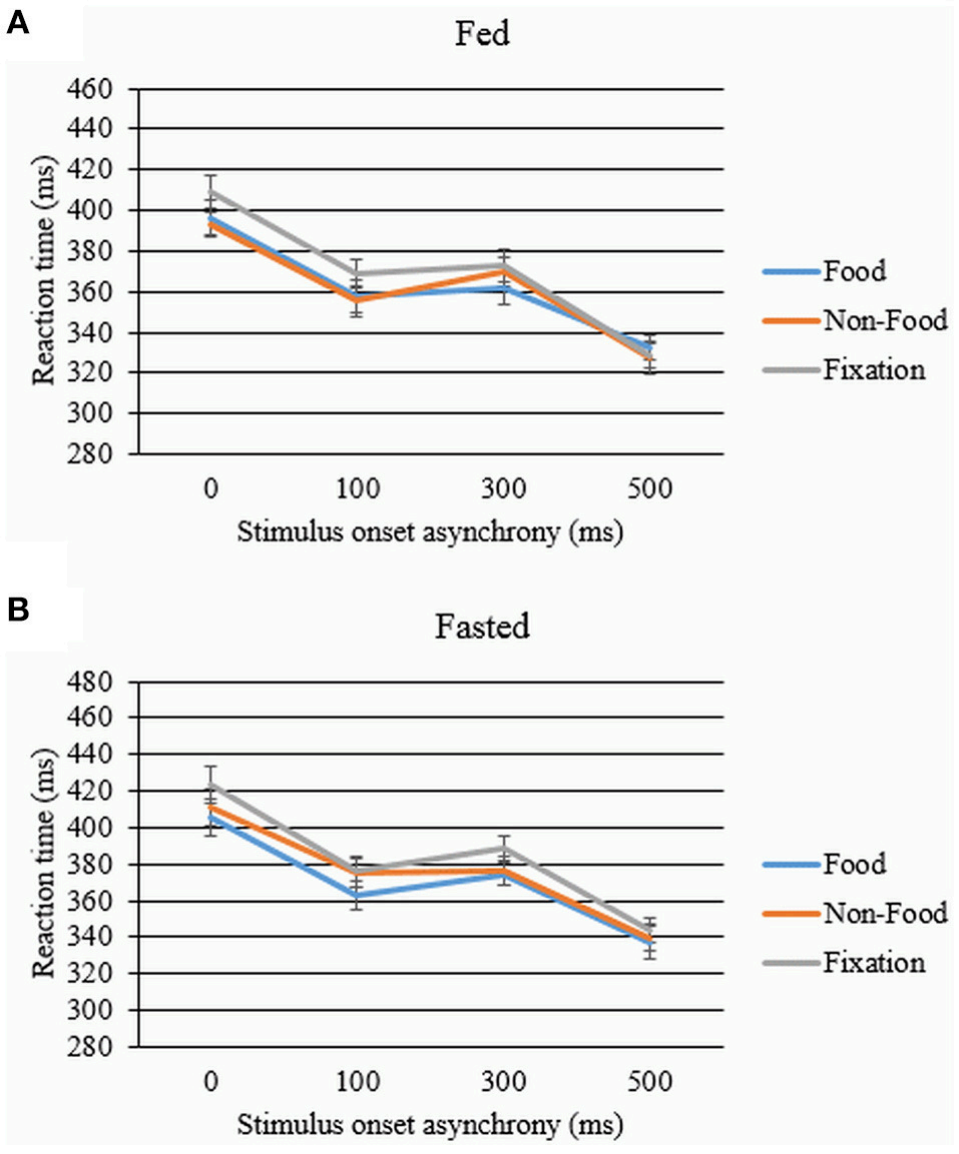
**Benefit of the warning cue in the (A) fed and (B) fasted states**. Error bars denote standard error (ms).

A 2 × 3 × 4 ANOVA was employed to assess the main effects and interactions between SOA, state and stimulus on reaction times in the mixed blocks. This revealed a significant main effect of stimulus [*F*_(1.677, 50.323^*^)_ = 17.277, *p* < 0.001] and SOA [*F*_(2.047, 61.409^*^)_ = 126.083, *p* < 0.001], but no main effect of state [*F*_(1, 30)_ = 2.728, *p* = 0.109]. No significant interactions were observed between state and stimulus [*F*_(2, 60)_ = 1.981, *p* = 0.147], state and SOA [*F*_(3, 90)_ = 0.071, *p* = 0.975], stimulus and SOA delay [*F*_(5.399, 161.973)_ = 1.438, *p* = 0.209] or between all three factors [*F*_(5.184, 155.514_^1^_)_ = 1.011, *p* = 0.415]. Bonferroni-corrected *post-hoc t*-tests revealed that participants responded significantly faster on food trials [*t*_(30)_ = −4.578, *p* < 0.001] and non-food trials [*t*_(30)_ = −4.762, *p* < 0.001] compared to fixation cross trials, with no difference between food and neutral trials [*t*_(30)_ = −1.474, *p* = 0.151]. Statistically significant differences were observed between all SOAs, which remained after correction was applied. Participants had significantly longer reaction times for the non-cued trials compared to cued trials with SOAs of 100 ms [*t*_(30)_ = 10.170, *p* < 0.001], 300 ms [*t*_(30)_ = 7.065, *p* < 0.001], and 500 ms [*t*_(30)_ = 14.226, *p* < 0.001]. Participants responded slowest at 500 ms compared to 100 ms SOA [*t*_(30)_ = 11.938, *p* < 0.001] and 300 ms SOA [*t*_(30)_ = 13.158, *p* < 0.001]. Unexpectedly, as can be seen in Figure 2, participants responded faster at 100 ms SOA compared to 300 ms SOA [*t*_(30)_ = −3.397, *p* = 0.012].

#### Effect of uncertainty in non-cued trials: preparation cost

**Figure 3:**
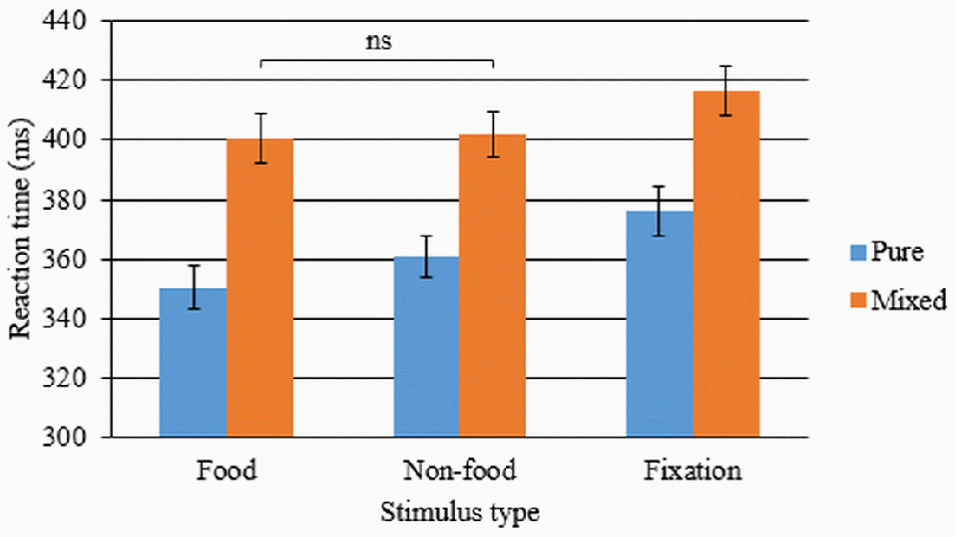
**Mean reaction times on non-cued food, non-food and fixation cross trials in the mixed and pure block of the proactive inhibition task**. Error bars denote standard error (ms). ns = not statistically significant comparison. All other within-condition comparisons remained significant after applying Bonferroni correction for multiple comparisons.

Reaction time during the pure block (only non-cued trials: no uncertainty) and the mixed block (mixture of cued and non-cued trials: some uncertainty) were compared using a 2 × 2 × 3 ANOVA to assess the impact of condition (pure/mixed), state (fed/fasted), and stimulus (food/non-food/fixation cross) on the employment of proactive inhibition as a goal-directed strategy (i.e., preparation cost). This yielded a significant main effect of condition, *F*_(1, 28)_ = 101.48, *p* < 0.001. Participants responded more slowly during the mixed block (mean RT = 406.35 ms, SD = 41.19 ms), which involved preparation for slowing responses to distinguish the target from the cue, compared to the pure block (mean RT = 365.39 ms, SD = 40.34 ms). *Post-hoc t*-tests revealed that participants responded faster in the pure block compared to non-cued trials within the mixed blocks for all stimulus types in the fed state [food: *t*_(28)_ = 7.064, *p* < 0.001; non-food: *t*_(28)_ = 9.425, *p* < 0.001; fixation cross: *t*_(28)_ = 6.677, *p* < 0.001] and fasted state [food: *t*_(28)_ = 7.270, *p* < 0.001; non-food: *t*_(28)_ = 7.657, *p* < 0.001; fix: *t*_(28)_ = 7.678, *p* < 0.001], all of which remained at *p* < 0.001 after Bonferroni correction. The ANOVA also revealed a main effect of stimulus type, *F*_(1.678,46.98^*^)_ = 39.612, *p* < 0.001, whereby average response time was fastest for food trials (mean = 380.69 ms, SD = 38.72 ms), followed by non-food trials (mean = 389.11 ms, SD = 39.58 ms) and slowest for fixation cross trials (mean = 395.29 ms, SD = 41.99 ms). These main effects were qualified by a significant interaction between condition and stimulus type [*F*_(1.748,48.954^*^)_ = 4.194, *p* = 0.025; see Figure 3]. *Post-hoc t*-tests revealed that this interaction was driven by significantly faster reaction times to food stimuli compared to neutral stimuli in pure [*t*_(28)_ = −5.222, *p*(corrected) < 0.001], but not the mixed block [*t*_(28)_ = −0.399, *p* = 0.693]. Response times remained significantly faster during food trials and neutral trials compared to fixation cross trials [all *t*_(28)_ ≥ 4.4, *p*(corrected) < 0.001]. In contrast, no main effect of state was observed, *F*_(1, 28)_ = 2.269, *p* = 0.143, nor any interactions between state and condition, *F*_(1, 28)_ = 1.498, *p* = 0.231, state and stimulus, *F*_(2, 56)_ = 1.237, *p* = 0.298, or all three factors, *F*_(2, 56)_ = 0.6, *p* = 0.553.

### Reactive inhibition

#### Index of inhibitory control: SSRT

Participants showed poorer inhibitory control in the fed (mean = 238.87, SD = 97.60 ms) compared to fasted (mean = 217.63 ms, SD = 90.48 ms) state, and to food cues (mean = 228.76 ms, SD = 102.10 ms) compared to non-food (mean = 217.17 ms, SD = 141.58 ms) and fixation cross cues (mean = 210.66 ms, SD = 126.18 ms). The means and standard deviations of the main SST outcomes (SSRT, mean RT, stop accuracy, go accuracy, and stop delay) for each stimulus in the fed and fasted states are presented in Table 3.

**Table 3:** **Means (and standard deviations) of outcome variables on the stop signal task**.

	**Fed**			**Fasted**					
	**Food**	**Non-food**	**No image**	**Food**	**Non-food**	**No image**	**ME state**	**ME stimulus**	**Interaction**
SSRT (ms)	236.74 (101.15)	250.89 (127.82)	228.99 (89.76)	225.51 (115.16)	212.65 (91.71)	220.98 (87.74)	*F*_(1, 44)_ = 5.665, *p* = 0.022	*F*_(2, 88)_ = 0.136, *p* = 0.873	*F*_(2, 88)_ = 2.437, *p* = 0.093
Mean RT (ms)	578.75 (233.76)	574.54 (241.62)	584.01 (241.17)	547.87 (225.93)	552.65 (212.03)	536.38 (202.41)	*F*_(1, 44)_ = 4.390, *p* = 0.042	*F*_(2, 88)_ = 0.086, *p* = 0.918	*F*_(2, 88)_ = 1.084, *p* = 0.343
Stop accuracy (%)	54.94 (22.94)	54.00 (25.80)	45.22 (20.72)	47.22 (20.10)	49.56 (22.58)	43.44 (19.06)	*F*_(1, 44)_ = 7.442, *p* = 0.009	*F*_(2, 88)_ = 11.546, *p* = 0.000	*F*_(2, 88)_ = 1.767, *p* = 0.177
Go accuracy (%)	98.95 (1.60)	98.75 (1.77)	98.70 (1.89)	99.08 (1.50)	98.83 (2.34)	98.41 (2.46)			
Stop delay (ms)	329.93 (189.97)	311.74 (186.28)	315.71 (190.25)	297.42 (172.27)	307.82 (174.96)	278.69 (167.65)			

#### SST main outcomes

The main outcomes of interest were the SSRT, mean RT, and stop accuracy in the fed and fasted state on each block. Repeated measures ANOVAs revealed a significant main effect of state on SSRT, mean RT, and stop accuracy. This was driven by poorer accuracy, faster reaction times, and smaller SSDs in the fed state (Table 3).

A main effect of stimulus was observed for stop accuracy but not for SSRT or mean RT. *Post-hoc t*-tests revealed this effect was driven by greater stop accuracy on food trials [mean = 51.08% (SD = 20.40%), *t*_(44)_ = 3.941, *p* = 0.001 (corrected)] and non-food trials [mean = 51.78% (SD = 22.06%), *t*_(44)_ = 3.764, *p* = 0.001 (corrected)] compared to no image trials (mean = 44.33%, SD = 18.33%), with no difference between food and non-food trials [*t*_(44)_ = −0.489, *p* > 0.05].

A trend toward a significant interaction between state and stimulus on SSRT was observed. Bonferroni-corrected *post-hoc t*-tests revealed that this interaction was driven by poorer inhibitory control (higher SSRT) to non-food cues in the fed compared to fasted state [*t*_(44)_ = 2.981, *p*(corrected) = 0.015]. No interaction between state and stimulus was observed for mean RT or stop accuracy.

### Correlations between inhibitory control and state measurements

Correlations were conducted to explore whether physiological state measurements were associated with self-reported mood, self-reported impulsivity and task-based measures of inhibitory control, whether self-reported impulsivity correlated with task-based impulsivity, and whether performance on different tasks correlated. As a main effect of state was observed for state measures (hunger, blood glucose) and some task-based measures of impulsivity [temporal discounting measures (Global discount factor, trend toward a main effect); stop signal task measures (stop accuracy, SSRT, mean RT)], correlations between these variables were calculated separately for the fed and fasted state. All other correlations were assessed across states.

#### Correlations between state measurements and inhibitory control

No associations were observed between state measurements (blood glucose, hunger) and assessments of either temporal discounting (global discount factor) in either state [fed (hunger: *r*_*s*_ = −0.077, *p* = 0.667; glucose: *r*_*s*_ = −0.205, *p* = 0.171); fasted (hunger: *r*_*s*_ = 0.067, *p* = 0.712; glucose: *r*_*s*_ = −0.138, *p* = 0.359)] or proactive inhibition [preparation cost (hunger: *r*_*s*_ = −0.182, *p* = 0.344; glucose: *r* = −0.167, *p* = 0.386), warning benefit (hunger: *r*_*s*_ = −0.065, *p* = 0.730; glucose: *r* = −0.067, *p* = 0.721)]. In contrast, while measures on the stop signal task were not related to state measurements in the fed state [mean RT (hunger: *r*_*s*_ = 0.120, *p* = 0.506; glucose: *r*_*s*_ = 0.106, *p* = 0.488), SSRT (hunger: *r*_*s*_ = 0_._103, *p* = 0.569; glucose: *r*_*s*_ = 0.079, *p* = 0.606), stop accuracy (hunger: *r*_*s*_ = 0.107, *p* = 0.554; glucose: *r* = 0.103, *p* = 0.500)], hunger statistically significantly negatively correlated with stop accuracy (%) (*r*_*s*_ = −0.348, *p* = 0.047) in the fasted state. Self-reported hunger and glucose were not correlated with any questionnaire measures of impulsivity [Delaying Gratification Inventory (hunger: *r*_*s*_ = −0.070, *p* = 0.705; glucose: *r* = −0.130, *p* = 0.395), Barratt Impulsiveness Scale motor subscale score (hunger: *r*_*s*_ = 0.243, *p* = 0.173; glucose: *r*_*s*_ = 0.135, *p* = 0.373)].

#### Correlations between task-based measures and questionnaire measures of inhibitory control and impulsivity

Total scores on the Delaying Gratification Inventory were significantly negatively correlated to Barratt Impulsiveness Scale motor subscale scores (*r*_*s*_ = −0.377, *p* = 0.011). Temporal discounting was related to both questionnaire measures: global discount factor positively correlated with Delaying Gratification Inventory total scores (trend: *r*_*s*_ = 0.287, *p* = 0.056) and negatively correlated with Barratt Impulsiveness Scale Motor subscale scores (*r*_*s*_ = −0.344, *p* = 0.019). With respect to proactive and reactive inhibition, few correlations with questionnaire measures of impulsivity were observed. Delaying Gratification Inventory total scores did not correlate with any outcome measure on the proactive inhibition task (preparation cost: *r*_*s*_ = −0.190, *p* = 0.332; warning benefit: *r*_*s*_ = 0.006, *p* = 0.976) or stop signal task (mean RT: *r*_*s*_ = −0.112, *p* = 0.468; SSRT: *r*_*s*_ = −0.101, *p* = 0.515; stop accuracy: *r*_*s*_ = −0.075, *p* = 0.626). Similarly, Barratt Impulsiveness Scale motor subscale scores did not correlate with outcome measures on the proactive inhibition task (preparation cost: *r*_*s*_ = −0.002, *p* = 0.991; warning benefit: *r*_*s*_ = −0.058, *p* = 0.758). While Barratt Impulsiveness Scale motor subscale scores were positively correlated with mean RT (trend: *r*_*s*_ = 0.258, *p* = 0.087) and SSRT (trend: *r*_*s*_ = 0.255, *p* = 0.091) on the stop signal task, it did not correlate with stop accuracy (%) (*r*_*s*_ = 0.245, *p* = 0.104).

Correlations between task outcome measures indicated a consistent positive relationship between outcomes on the proactive inhibition and stop signal tasks. Warning benefit was significantly positively correlated with mean RT (*r*_*s*_ ≤ −0.451, *p* = 0.012), SSRT (*r*_*s*_ ≤ 0.394, *p* = 0.031), and stop accuracy (*r*_*s*_ ≤ 0.367, *p* = 0.046) on the stop signal task. In contrast, preparation cost did not correlate with any stop signal task measure (all *r*_*s*_ ≤ 0.095, *p* ≥ 0.630). No correlations were observed between temporal discounting and proactive inhibition task outcomes (all *r*_*s*_ ≤ −0.066, *p* ≥ 0.725) or between temporal discounting and stop signal task outcomes in either state (all *r*_*s*_ ≤ −0.168, *p* ≥ 0.173).

## Discussion

This study examined the influence of promoting a “need” state (via acute food restriction) and motivationally relevant distraction (food images) on several aspects of inhibitory control. It was predicted that inhibitory control is impaired after food restriction, i.e., in the fasted compared to the fed state; that food stimuli will promote approach behaviors leading to poorer inhibitory control, and that food images will impair inhibitory control most strongly in the fasted condition due to motivational relevance.

### State effects

Our data partially supported our hypothesis that acute food restriction would impair inhibitory control, as participants exhibited greater temporal discounting (preference for smaller immediate rewards) after acute fasting. However, food restriction had no effect on questionnaire measures of inhibitory control or on proactive inhibition. Moreover, reactive inhibitory control on the SST in fact improved after fasting.

Contrary to De Ridder et al. (2014), our participants showed greater temporal discounting after acute food restriction compared to when satiated. This difference in findings may be due to the methods used in the two studies: we employed a repeated-measures design, whereas a between-subjects design was used by De Ridder et al. (2014). In addition, hunger did not correlate with temporal discounting in either state. In contrast, our data revealed improved reactive inhibitory control (lower SSRT and greater stop accuracy) in the fasted state compared to the fed state. However, this may be influenced by level of hunger, as hunger negatively correlated with stop accuracy in the fasted state. In other words, individuals who were less hungry after acute fasting showed greater stop accuracy than those who were more hungry after acute fasting.

These findings hold implications for individuals who engage in dieting and food restraint, as well as for individuals with eating disorders. The observation that acute food restriction leads to poorer inhibitory control performance may provide some explanation as to why poorer inhibitory control is often reported in restrained eaters and has been reported in eating disorders (for review, see Bartholdy et al., 2016). As eating restraint was not assessed in this study, it is of interest to explore whether this is the case in restrained eaters and in successful and unsuccessful dieters to understand how inhibitory control in the context of various eating patterns/behavior is affected by food and hunger, and how this might translate into success or failure in dietary restraint, weight management and overall healthy and unhealthy eating patterns. Future studies may also wish to explore the impact of food-specific inhibitory control on later consumption of food to assess disinhibition effects in healthy non-dieters.

### Motivational relevance

Stimulus type had an effect on proactive preparation cost and the accuracy of reactive inhibition (stop accuracy). When comparing the reaction time of non-cued trials in the pure and mixed blocks on the proactive task, a significant main effect of stimulus was observed, with overall faster reaction times on food trials (compared to non-food and fixation cross trials). This is in line with previous studies that have revealed faster approach behaviors to food stimuli (e.g., Seibt et al., 2007). Moreover, an interaction between stimulus and condition was observed: reaction times were significantly shorter on food trials than both non-food and fixation cross trials in the pure block, but were not significantly shorter than non-food trials in the mixed block. As the mixed block was associated with increased uncertainty (i.e., in terms of whether or not the upcoming visual stimulus was a target or a warning signal), this finding may reflect either additional effortful slowing in the context of food (i.e., greater inhibition of approach motivated behaviors) to ensure accurate responding, or that food stimuli facilitated approach behaviors but only in the context of reduced uncertainty. Alternatively, improved performance on food trials may reflect alertness or arousal, i.e., individuals may be more alert to food stimuli when hungry.

The data also revealed a main effect of stimulus on stop accuracy, driven by poorer inhibition accuracy during no image trials. This is in contrast to our hypotheses as it was predicted that inhibitory control performance would be worse on food trials as food is thought to stimulate approach behaviors. However, it may have been that the presence of the distracting images had a facilitatory effect on performance. Both the arrow (target) and the distractors were removed from the screen and replaced by the stop signal during stop trials. This may have directed their attention away from the point/target of fixation which may have altered the detection of the stop signal. This is unlikely to fully explain this effect, as the arrow duration was only slightly higher than the mean SSDs across stimulus conditions and states, meaning this would have only provided a facilitatory effect at very short SSDs.

Although not assessed in this study, external eating may have contributed to the observed effects of stimulus type on inhibitory control. External eating refers to the tendency to overeat in response to food cues, and is thought to be associated with both enhanced attentional bias to food cues and increased trait impulsivity (Brignell et al., 2009; Nijs et al., 2009; Hou et al., 2011). Thus, food stimuli may be particularly salient to external eaters, and therefore may have more of an influence on their ability to control their behavior compared to non-external eaters, relative to non-food stimuli. It would be of interest to explore the potential mediating role on inhibitory control in the context of food cues, and also how external eating may be influenced by state. Further research may wish to explore external eating by incorporating taste-tests in the laboratory (in which participants are invited to eat as much as they wish of the foods provided) to assess differences in subsequent food intake after exposure to food stimuli compared to non-food stimuli. Such research will improve our understanding of the mechanisms underlying the relationship between food stimuli and eating behaviors in non-clinical populations. It will also provide useful insight into the contexts in which inhibitory control is most influenced by external eating, and therefore the contexts in which behavioral training paradigms may be most effective.

### Relation of inhibitory control to physiological measures

Our data revealed some evidence for a relationship between questionnaire or task-based measures of inhibitory control and physiological measures of hunger and glucose. Despite the notion that self-control is a stable predisposition over time (Davis et al., 2010), previous research has shown that blood glucose levels have an effect on executive function (Stephens and Tunney, 2004) and self-regulation. For example, one study revealed a negative relationship between temporal discounting and blood glucose (manipulated via drinks containing either sugar or sweetener): individuals with lower blood glucose levels showed a higher degree of temporal discounting (i.e., a greater inclination to choose smaller immediate rewards than larger, delayed rewards), whereas individuals with increased blood glucose after ingestion of the sugar-containing drink showed a greater valuation of future rewards (Wang and Dvorak, 2010). Reducing fluctuations in blood glucose levels has even been suggested as a means of improving temporal discounting in disorders characterized by impulsive or compulsive behaviors, such as eating disorders (Wang and Dvorak, 2010). With respect to motor inhibition, evidence from the literature on eating disorders suggests poorer inhibitory control (i.e., longer SSRTs) on the stop signal task in binge-related eating disorders, which are associated with largely fluctuating glucose levels (for review, see Bartholdy et al., 2016).

Contrary to these previous findings, the present data did not reveal a relationship between glucose and task-based measures of temporal discounting, proactive inhibition, or reactive inhibition, which may indicate that state-related differences in performance were not underscored by physical differences in energy levels. The discrepancy between our data and previous temporal discounting findings may be related to differences in the procedure for manipulating blood glucose: our study involved an overnight fast and thus may reflect differences in resting glucose levels, whereas Wang and Dvorak (2010) manipulated their participants' blood glucose level through consumption of a soda drink containing either sugar or artificial sweetener. With respect to self-reported inhibitory control, our data revealed a trend toward a negative correlation between Delaying Gratification Inventory total scores and glucose, and a positive correlation between glucose and self-reported motor impulsivity on the Barratt Impulsiveness Scale, in the fasted but not fed state. Together our findings suggest that lower glucose levels are associated with greater self-reported inhibitory control in the fasted state but are not related to inhibitory control assessed using neuropsychological tasks.

### Methodological considerations

This study benefited from its repeated measures design, inclusion of both subjective and objective measurements of inhibitory control and state (glucose, self-reported hunger, and eating), and by assessing several different types of inhibitory control. In addition, this study is the first to explicitly discuss proactive inhibition in the context of eating behavior (hunger state), and findings from this task can form the basis for future studies assessing proactive inhibition in eating disorders and obesity.

This study has several limitations. With respect to paradigm design, the images in the stop signal task may have also facilitated go performance accuracy as they provided additional visual information that may have altered attention and subsequent detection of the stop signal. However, go accuracy was high across all conditions, thus the task was already sufficiently simple and any facilitation may be a small effect. In the temporal discounting task, only one time delay was assessed (3 months). Although we used a greater number of trials than typical designs that employ hyperbolic modeling of intertemporal choice behavior, future studies may wish to explore a range of delays to obtain a more comprehensive assessment of participants' valuation of monetary rewards over time. In addition, the impact of stimuli in the SST or proactive inhibition task may be more reliably distinguished using a blocked design in which stimuli are presented in different blocks to remove any lingering effects of previous stimuli on behavior in subsequent trials.

Other limitations relate to confounding factors and generalizability. Potential confounding factors such as income and level of education were not assessed. Although this study was only assessing within-subjects factors, and thus the impact of these confounds on the present results should in theory be minimal, this may limit the generalizability of our findings to populations similar in age and education to our sample. While current dieters were excluded during screening, eating restraint tendencies were not assessed in our sample. This is important as evidence suggests that restrained eaters have poorer inhibitory control compared to unrestrained eaters (Bartholdy et al., 2016). Future studies may wish to explore whether inhibitory control in restrained eaters is differentially affected by state or stimuli compared to unrestrained eaters. Additionally, our sample was predominantly comprised of women. While there is some evidence to suggest gender differences in inhibitory control and impulsivity in adults and children, a meta-analysis of healthy adults reported that these gender differences are most evident with respect to reward and punishment sensitivity, risk taking and sensation seeking, whereas no differences between men and women were observed on tasks involving effortful inhibitory control, including assessments of reactive inhibition (go/no-go task, stop signal task), reward-based inhibition (temporal discounting), or cognitive inhibition (e.g., the Stroop task) (Cross et al., 2011). However, future studies may wish to include a more even distribution of men and women to control for possible effects of gender in their analyses.

## Conclusions

The lack of correlations between inhibitory control measures in this study supports the notion that these neuropsychological tasks and questionnaires are tapping into distinct subcomponents of inhibitory control. Acute food restriction did not influence questionnaire measures of inhibitory control, but did appear to affect reactive and reward-based inhibitory control performance on neuropsychological tasks. Participants were on the whole faster at responding to food images in the proactive task, suggesting that food stimuli do motivate approach behavior. However, state did not interact with motivational relevance of distracting stimuli, suggesting that stimulus relevance does not have any additive influences over inhibitory control in non-dieting healthy individuals. Future studies may wish to replicate this study in external eaters, restrained eaters and current dieters to explore whether the influence of food restriction on an individual's ability to exhibit inhibitory control is associated with their responsiveness to food cues, success of eating restraint or weight regulation.

## Author contributions

SB and OO conceived and designed the study. SB and JC collected the data. SB, JC, and OO analyzed the data. SB drafted the manuscript. JC, OO, IC, and US critically revised the manuscript. All authors were involved in *interpretation* of the data, drafting, critiquing and approving the manuscript and accept responsibility for the accuracy and integrity of this work.

### Conflict of interest statement

The authors declare that the research was conducted in the absence of any commercial or financial relationships that could be construed as a potential conflict of interest.

## Footnotes

^1^Due to violation of the assumption of sphericity, Huynh Feldt degrees of freedom and *p* values are reported.
